# Influence of meteorological and environmental factors on pediatric urinary tract infections: insights from a 6-year retrospective study in Central China

**DOI:** 10.3389/fpubh.2025.1512403

**Published:** 2025-02-13

**Authors:** Changzhen Li, Feng Tang, Lei Xi, Xiaomei Wang

**Affiliations:** Wuhan Children’s Hospital (Wuhan Maternal and Child Healthcare Hospital), Tongji Medical College, Huazhong University of Science & Technology, Wuhan, China

**Keywords:** pediatric urinary tract infections, uropathogens, distribution characteristics, meteorological factors, antibiotic resistance, retrospective study

## Abstract

**Objectives:**

To investigate the association between meteorological factors and common uropathogens in children with urinary tract infections (UTIs) and assesses the potential influence of weather conditions on pediatric UTIs.

**Study design:**

Analyze the demographic and uropathogen characteristics from children with culture-proven UTIs and its correlation with meteorological factors.

**Methods:**

2,411 data from infants and children with UTIs in a children’s hospital from 2016 to 2021 were retrospectively analyzed. A correlation analysis was conducted to investigate the relationship between the monthly detection number of uropathogens and meteorological factors.

**Results:**

Multiple linear stepwise regression analyses showed a positive correlation between monthly average temperature, precipitation volume, sunshine hours, monthly total number of uropathogens, and the number of *E. coli* and *E. faecalis*. *E. faecium* was predominant in <12-month-old children, while *E. coli* was dominant in the 3–18-year age category. *E. faecium* showed a higher prevalence in girls, while *E. faecalis* was more prevalent in boys. *E. coli* exhibited resistance rates of >40% to second-or third-generation cephalosporins in multiple age groups. *E. faecium* showed high resistance rates to tetracyclines, fluoroquinolones, erythromycin, ampicillin, and penicillin, while *K. pneumoniae* displayed higher sensitivity to cephalosporin–sulbactam and amikacin, but higher resistance rates to cefazolin and ceftazidime.

**Conclusion:**

This study reveals the association between meteorological factors and uropathogens in children with UTIs, as well as the distribution, age-related characteristics, gender differences and antibiotic resistance profiles of pathogenic bacteria. These findings inform the development of targeted strategies for UTI prevention and treatment based on uropathogenic characteristics and meteorological conditions.

## 1 Introduction

Urinary tract infections (UTIs) are a common infectious disease characterized by the invasion of pathogens into the urinary tract, including the urethra, bladder, and kidneys (1). They are the second most common infection worldwide, contributing to approximately 20–60% of all infections and significantly impacting morbidity and mortality in both outpatient and inpatient settings, with an estimated 7% of girls and 2% of boys developing at least one UTI by age 6 (2). UTIs are common worldwide and pose a significant burden on the health and quality of life of the individual, as well as the economy of countries (3). Among the various populations, UTIs in children have attracted widespread attention and research efforts (4). UTIs are relatively common in children, particularly in infants and preschool-aged children. It has been reported that children and adolescents with lower urinary tract dysfunction are 2.6 times more likely than those with normal urinary function to develop emotional and behavioral problems (5). Therefore, understanding the epidemiology of UTIs in children is of paramount importance.

The incidence of UTIs in children varies based on age, gender, and population differences. Girls are more susceptible to UTIs than boys, which may be attributed to physiological factors such as a shorter urethra and closer proximity of the urethral opening to the anus in girls (4). This anatomical structure of the female urinary tract predisposes girls to bacterial seeding and the proximal spread of pathogens from the perineum to the urinary tract. The shorter urethra increases the frequency of infections, particularly in younger children. Additionally, the colonization of the perineum by dominant fecal flora, including coliform bacteria, is thought to play a role in the ascent of bacteria to the urinary tract, further elevating the risk of UTIs in girls (6). Additionally, some young girls experience recurrent episodes of cystitis or pyelonephritis, which contribute to their increased vulnerability to UTIs (7). The immature immune system of children, combined with nutrient deficiencies, particularly vitamin D, plays a significant role in their increased susceptibility to infections such as UTIs (8). Vitamin D is critical for maintaining immune health, and its deficiency—especially in regions like Wuhan, where vitamin D deficiency rates are high (9)—can significantly increase the risk of UTIs in pediatric populations.

Understanding the distribution of pathogens, age-related characteristics, and antibiotic resistance is crucial for the management and understanding of pediatric UTIs (10). Although previous studies have reported on the prevalence of different pathogens that cause UTIs in children, there is still a lack of a deeper understanding of their age-related characteristics and antibiotic resistance (11, 12). In particular, the age-specific distribution of pathogens and the variations in their resistance profiles across different pediatric age groups have not been thoroughly explored. It is unclear whether there are significant differences in the dominant pathogens between infants and older children. Moreover, the variation in antibiotic resistance across age groups has not been adequately studied, especially regarding common pathogens such as *E. coli*, where resistance patterns may differ between infants and older children. This knowledge is crucial for developing individualized treatment strategies. Furthermore, the relationship between meteorological factors and bacterial infections is attracting an increasing research interest. Evidence suggests that meteorological factors influence the transmission of bacterial dysentery (13). Climatic factors, particularly temperature, influence the growth, survival, and transmission pathways of pathogens, thereby affecting the spread of infectious diseases (14). Previous studies have explored the impact of meteorological conditions on UTIs incidence. In adults, studies have shown significant positive correlations between meteorological variables like average monthly temperature, sunshine hours, precipitation, and rainfall days with the incidence of female pyelonephritis (15). Additionally, rising temperatures have been linked to a higher rate of outpatient UTIs, particularly during the shoulder seasons of spring and autumn (16). However, while these associations have been established in adult populations, the impact of meteorological factors on UTI incidence in children remains largely unexplored. Specifically, it is unclear whether children experience similar relationships between meteorological factors and UTI incidence. Additionally, there is a lack of research on the correlation between specific pathogens causing UTIs and meteorological factors. Our study fills this gap by investigating the correlation between meteorological factors and pathogenic bacterial infections in pediatric UTIs, building upon a comprehensive understanding of the distribution of pathogens, age-related characteristics, and antibiotic resistance.

By studying patients of different ages and genders, we aim to gain further insights into the distribution and characteristics of pathogens in these specific populations, providing more accurate evidence that would enable individualized treatment and management. Additionally, through this research, we aim to uncover the prevalence of pediatric UTIs and their potential relationship with climatic factors, providing a deeper theoretical foundation and clinical guidance for the prevention and treatment of pediatric UTIs.

## 2 Methods

### 2.1 Study population

**Figure 1:**
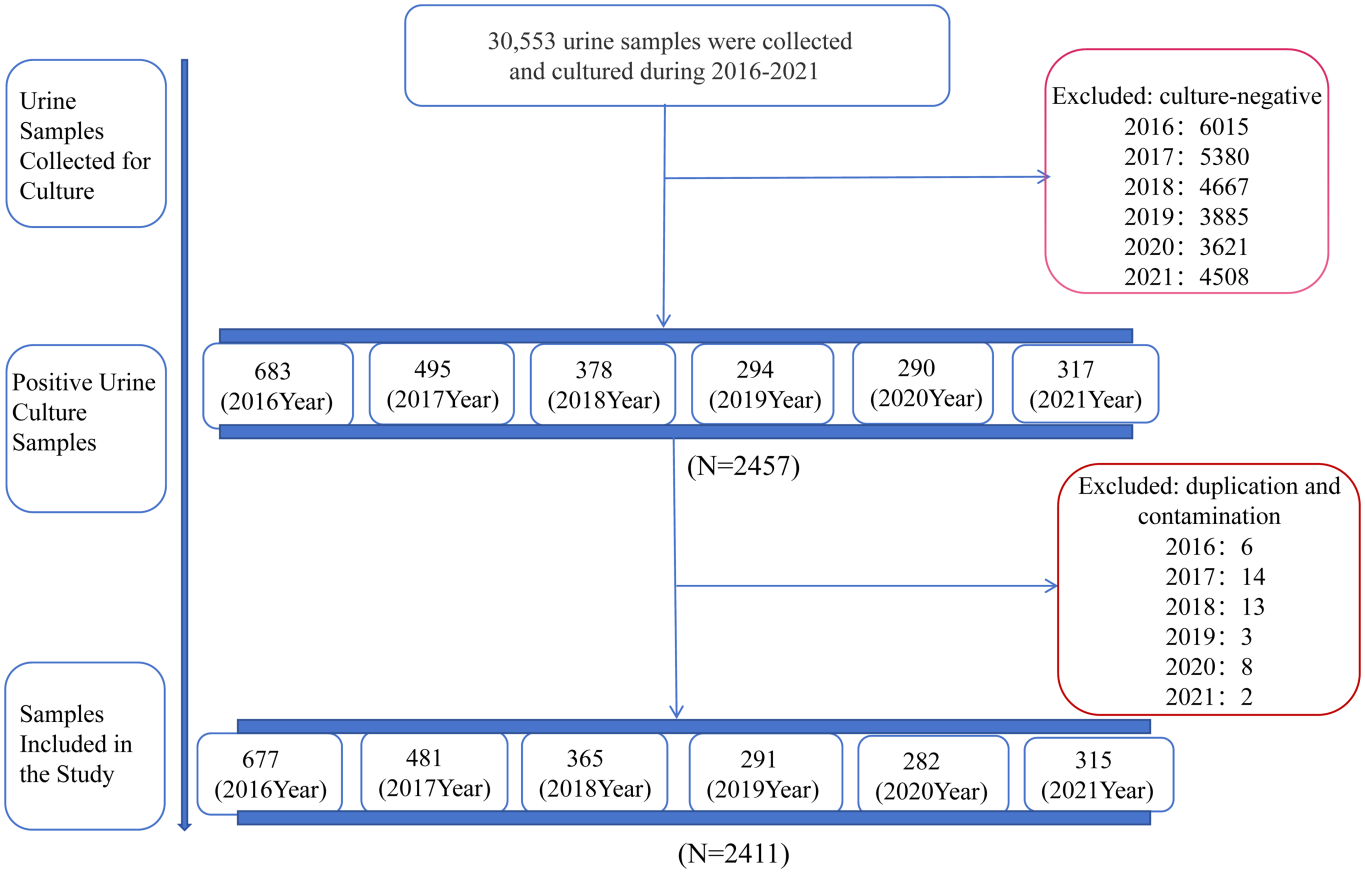
Flowchart of the selection process of the study population.

From 2016 to 2021, 30,553 urine culture samples were collected from pediatric patients in a children’s hospital in central China. The inclusion criteria were pediatric patients with suspected UTIs based on their symptoms and pyuria in routine urinalysis. The exclusion criteria were as follows: (1) culture-negative specimens; (2) multiple culture results from the same patient during hospitalization; and (3) contamination, defined as the presence of more than three isolates of the same organism (17). As a result, 28,076 culture-negative samples and 46 isolates from patients with multiple culture results or contamination were excluded from the analysis, ensuring the validity of the study (Figure 1). For urine sample collection in children, we employ stimulation of voiding techniques, urethral catheterization or suprapubic sampling for non-toilet-trained infants, while toilet-trained children are asked to provide a midstream clean-catch urine sample (18). To explore the potential association between meteorological factors and specific pathogens causing UTIs, we narrowed down our study population to pediatric UTIs caused by a single pathogen species (*N* = 2,411).

### 2.2 Definitions

We defined UTI by the presence of all the following 3 criteria: (1) 1 or more of the following signs or symptoms of UTI: fever (defined as temperature of at least 38°C); pain in the suprapubic, abdominal, or flank area; urinary urgency, frequency, or hesitancy; dysuria in children 2 years or older or in children younger than 2 years of age; and poor feeding or vomiting; (2) pyuria, defined as 10 or more white blood cells per cubic millimeter (uncentrifuged specimen) or 5 or more white blood cells/high-powered field (centrifuged specimen) or leukocyte esterase more than or equal to trace on dipstick urinalysis; (3) a positive urine culture defined by growth of a single uropathogen at counts 5 × 10^4^ or higher CFU per mL (suprapubic aspiration or catheterized specimen) or 10^5^ or higher CFU per mL (clean voided specimen) (19).

### 2.3 Strain identification and antibiotic susceptibility tests

The isolates were identified using matrix-assisted laser desorption ionization-time of flight mass spectrometry (MALDI-TOF MS; Bruker, Germany), supplemented with traditional methods such as biochemical testing, serotyping, and genetic analysis, particularly when MALDI-TOF MS alone could not provide conclusive identification. Biochemical testing was supported by VITEK2 Compact (bioMérieux, France), and genetic analysis was employed for further confirmation when necessary (20). All identified strains were stored in skim milk agar stock at −80°C for further analysis. Antibiotic susceptibility tests were performed using the VITEK 2 Compact automated system (bioMérieux, France) in accordance with the guidelines of the Clinical and Laboratory Standards Institute (CLSI) as well as its Advanced Expert System (AES). Briefly, a 0.5 McFarland standard bacterial suspension of isolates was prepared, dispensed into the intended plates (GP68, GP67, and GN335 plate, bioMérieux, France), which was pre-encapsulated with intended antibiotics of two-fold serial dilutions. MIC concentrations were determined and the results were categorized as resistant, intermediate, or susceptible according to MIC breakpoints recommended by the CLSI. Quality control strains, including *E. coli* ATCC 25922, *S. aureus* ATCC 25923, and *P. aeruginosa* ATCC 27853, were used.

### 2.4 Data on meteorological factors

In Wuhan, the city is divided into 13 administrative districts, each containing a total of 176 environmental monitoring stations. Since the majority of samples from hospitalized patients were collected from these districts, we utilized averaged data from all monitoring stations as the city-wide environmental indicators, with outliers excluded based on Grubbs’ test. Data on monthly meteorological factors from 2016 to 2021 were obtained from the publicly accessible China National Meteorological data sharing system. Meteorological factor variables, such as average temperature (°C), relative humidity (%), average volume of precipitation (mm), and sunlight hours (h), were used.

### 2.5 Statistical analysis

Statistical analysis was performed using SPSS 24.0 software. Categorical variables were analyzed by *χ*^2^-test or Fisher’s exact test. OriginLab version 2021 (OriginLab Corporation, Northampton, MA, USA) was used to perform the analyses and draw the figures (pathogen distribution diagram and antimicrobial resistance rates figures). To address the potential issue of multicollinearity, Multiple linear stepwise regression analysis was conducted to explore the relationships between the monthly detection of major uropathogens and meteorological factors. Multicollinearity was assessed by examining tolerance and the variance inflation factor (VIF). Tolerance values below 0.10 or VIF values above 10 were indicative of multicollinearity. A *p*-value of less than 0.05 (two-tailed) was considered statistically significant for interpreting the results (21). Additionally, to control for potential confounding factors that may influence the relationship between meteorological factors and pathogen distribution, we employed several adjustment strategies. Specifically, demographic variables such as age, sex, and underlying health conditions (e.g., urinary tract malformations, renal cysts, immunodeficiencies) were included as covariates in the regression models. By adjusting for these factors, we aimed to isolate the specific influence of meteorological factors on the incidence of uropathogens in pediatric UTIs. This approach ensures that the observed relationships reflect the true effects of environmental factors, rather than being confounded by individual characteristics or health conditions.

## 3 Results

### 3.1 Distribution of the main uropathogens

A total of 30,553 urine samples were analyzed. Note that 2,411 strains of pathogenic bacteria were isolated from all midstream urine samples, resulting in a positive rate of 7.9%, with the following yearly distribution: 677 in 2016, 481 in 2017, 365 in 2018, 291 in 2019, 282 in 2020, and 315 in 2021. The age group of 29 days to 6 months exhibited the highest positive detection rate, while no significant difference was observed in the positive rates between different genders (Table 1).

**Table 1:** Gender and age group distribution of the study population from 2016 to 2021.

Parameter	2016 *n*(%)	2017 *n*(%)	2018 *n*(%)	2019 *n*(%)	2020 *n*(%)	2021 *n*(%)	Total *N*(%)
Gender							
Male	360 (53.2%)	235 (48.9%)	161 (44.1%)	127 (43.6%)	150 (53.2%)	157 (49.8%)	1,190 (49.4%)
Female	317 (46.8%)	246 (51.1%)	204 (55.9%)	164 (56.4%)	132 (46.8%)	158 (50.2%)	1,221 (50.6%)
Age							
≤28 d	17 (2.5%)	25 (5.2%)	28 (7.7%)	14 (4.8%)	18 (6.4%)	12 (3.8%)	114 (4.7%)
29 d–6 months	244 (36.0%)	111 (23.1%)	125 (34.2%)	101 (34.7%)	114 (40.4%)	104 (33.0%)	799 (33.1%)
6–12 months	118 (17.5%)	54 (11.2%)	66 (18.1%)	53 (18.2%)	45 (16.0%)	72 (22.9%)	408 (16.9%)
1–3 years	105 (15.5%)	122 (25.4%)	71 (19.5%)	50 (17.2%)	33 (11.7%)	47 (14.9%)	428 (17.8%)
3–6 years	98 (14.5%)	79 (16.4%)	38 (10.4%)	37 (12.7%)	39 (13.8%)	37 (11.7%)	328 (13.6%)
6–18 years	95 (14.0%)	90 (18.7%)	37 (10.1%)	36 (12.4%)	33 (11.7%)	43 (13.7%)	334 (13.9%)
Total *N*(%)	677 (28.1%)	481 (19.6%)	365 (15.1%)	291 (12.1%)	282 (11.7%)	315 (13.1%)	2,411 (100%)

Percentages are calculated within each year and total column based on the total population for that specific category. Absolute numbers (n) and percentages (%) are presented for each category.

**Figure 2:**
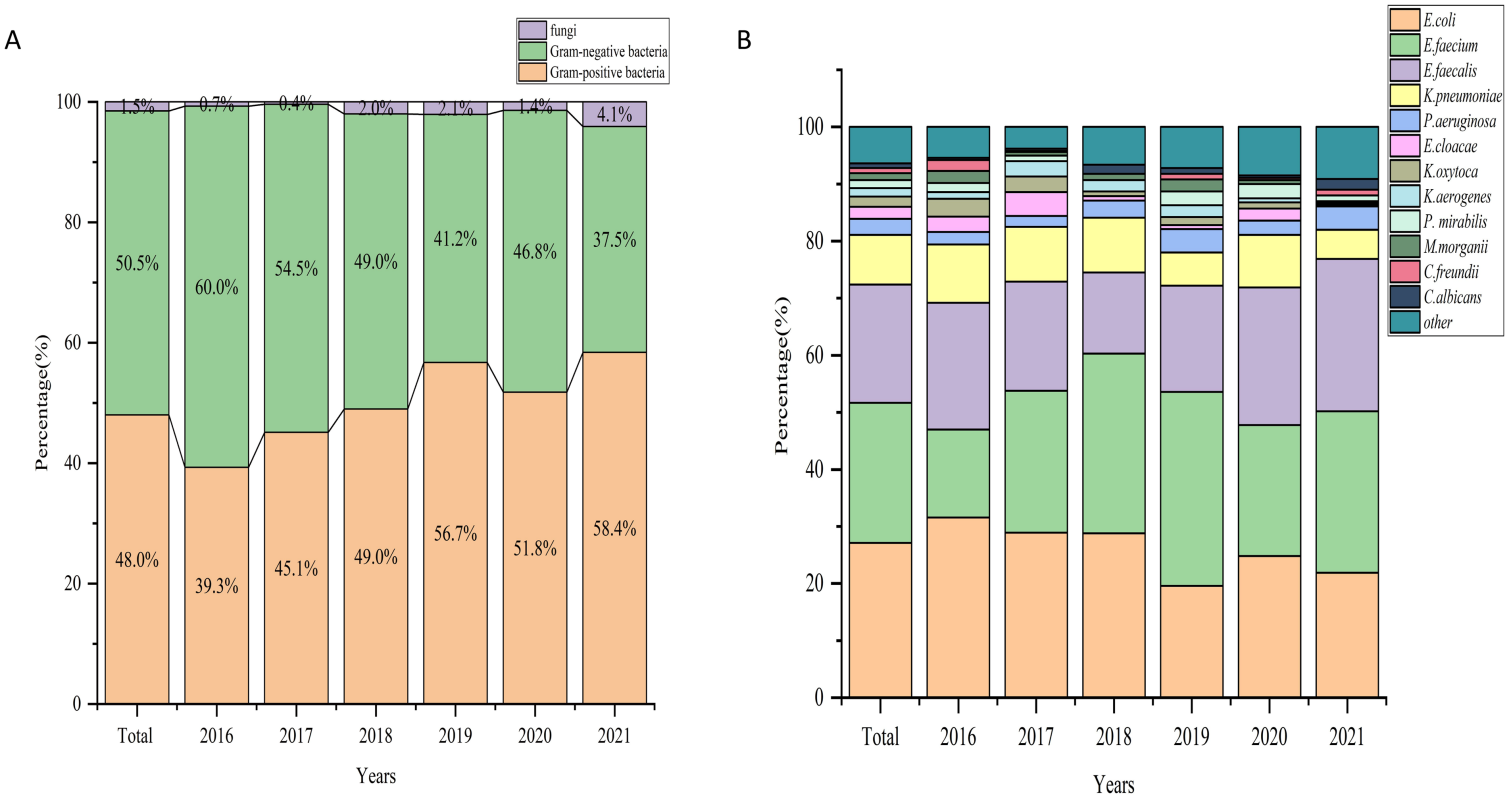
**(A)** Percentage distribution of gram-positive, gram-negative, and fungal pathogens causing UTIs across years (2016–2021). **(B)** Percentage distribution of major bacterial pathogens causing UTIs across years (2016–2021).

Among all the positive results, Gram-negative bacteria accounted for 50.5% (1,217), Gram-positive bacteria for 48.0% (1,157), and fungi for 1.5% (37) of the isolated strains (Figure 2A). The top 10 pathogenic bacteria were *E. coli* (27.1%), *E. faecium* (24.6%), *E. faecalis* (20.7%), *K. pneumoniae* (8.7%), *P. aeruginosa* (2.8%), *E. cloacae* (2.1%), *K. oxytoca* (1.8%), *K. aerogenes* (1.5%), *Proteus mirabilis* (1.4%), and *M. morganii* (1.2%). Among the Gram-positive bacteria, *E. faecium* showed a significant increase in the detection rate, from 15.4% in 2016 to 28.3% in 2021. *E. faecalis* also exhibited some fluctuations, although it maintained a consistent detection rate, reaching 26.7% in 2021. In contrast, Gram-negative bacteria, such as *E. coli*, demonstrated a gradual decline in the detection rate, decreasing from 31.6% in 2016 to 21.9% in 2021. *K. pneumoniae*, another common Gram-negative species, remained relatively stable with a detection rate of 5.1% in 2021. Other bacteria, including *P. aeruginosa, E. cloacae*, and *K. oxytoca*, showed varying detection rates without significant overall trends (Figure 2B).

### 3.2 Age distribution prevalence characteristics of the main uropathogens

**Figure 3:**
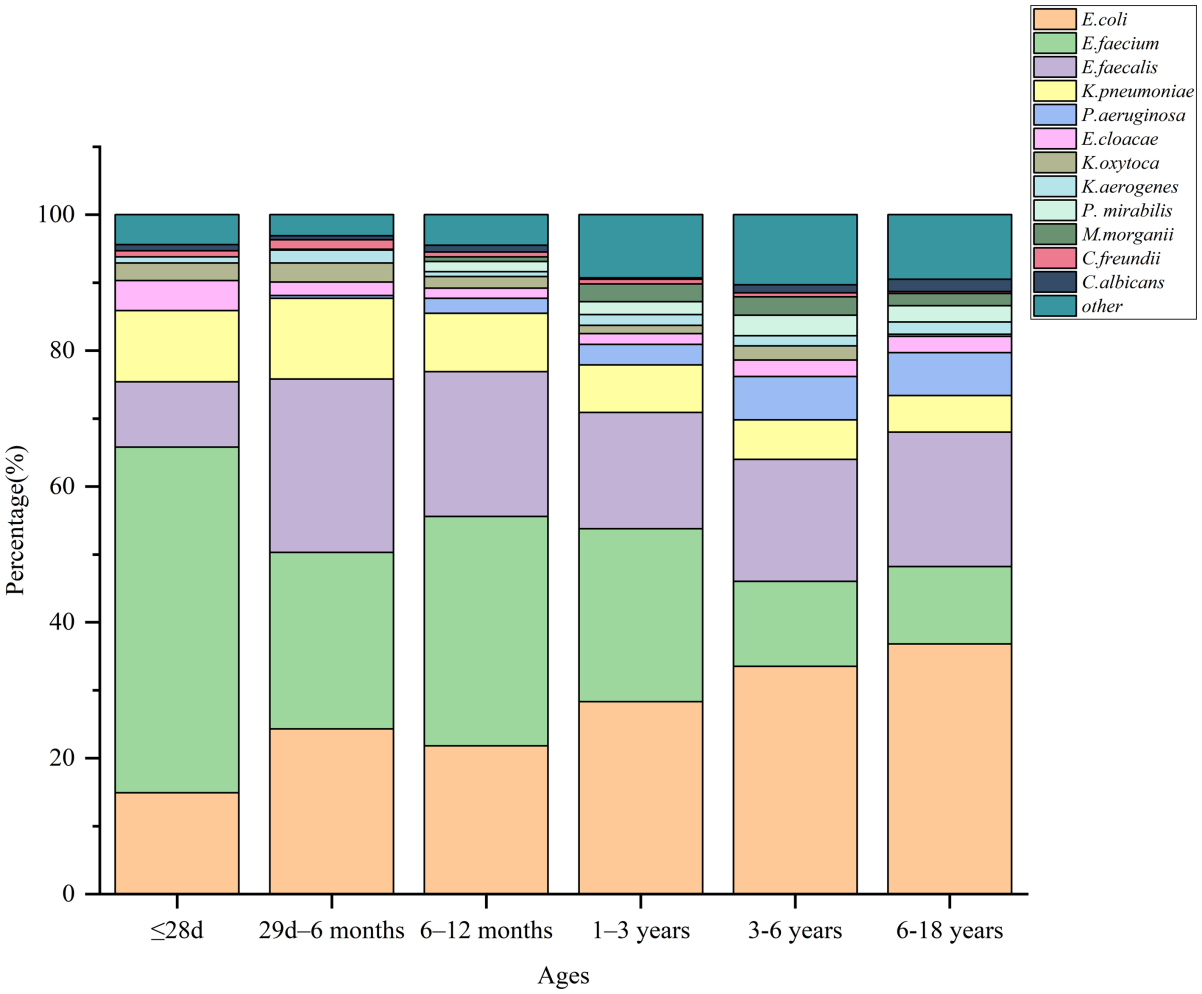
Distribution of major urinary pathogens in different age categories.

Figure 3 presents the comprehensive etiological distribution across various age groups. *E. faecium* was predominant in the neonatal group (50.9%, *N* = 58), the 29-day–6-month group (26.0%, *N* = 208), and the 6–12-month group (33.8%, *N* = 138). Conversely, *E. coli* was the dominant pathogen in the 3–6-year age category (33.5%, *N* = 110) and the 6–18-year age category (36.8%, *N* = 123).

### 3.3 Distribution of main uropathogens in relation to gender

Supplementary Table 1 presents the distribution of the main uropathogens in relation to gender. The prevalence of *E. coli* was similar between girls (27.8%) and boys (26.5%) (*p* = 0.452). However, significant gender differences were observed for other pathogens. *E. faecium* showed a higher prevalence in girls (31.8%) compared to that in boys (17.1%) (*p* < 0.01). In contrast, *E. faecalis* was more prevalent in boys (23.1%) than it was in girls (18.4%) (*p* < 0.05). *K. aerogenes*, *P. mirabilis*, and *M. morganii* were also found to have significantly higher prevalence in boys than they had in girls (*p* < 0.05). However, no significant gender differences were observed for *K. pneumoniae*, *P. aeruginosa*, *E. cloacae*, and *K. oxytoca*. The etiological profile of boys and girls of different age groups is shown in Supplementary Table 1. The prevalence of *E. coli* exhibited an increasing trend in girls with advancing age, while it remained stable in boys. Notably, *E. faecium* was the predominant pathogen in newborns of both genders, accounting for 46.3% of cases in boys and 55% in girls (Supplementary Table 2).

### 3.4 Correlation between monthly detection of the main uropathogens and monthly mean values of meteorological factors

**Figure 4:**
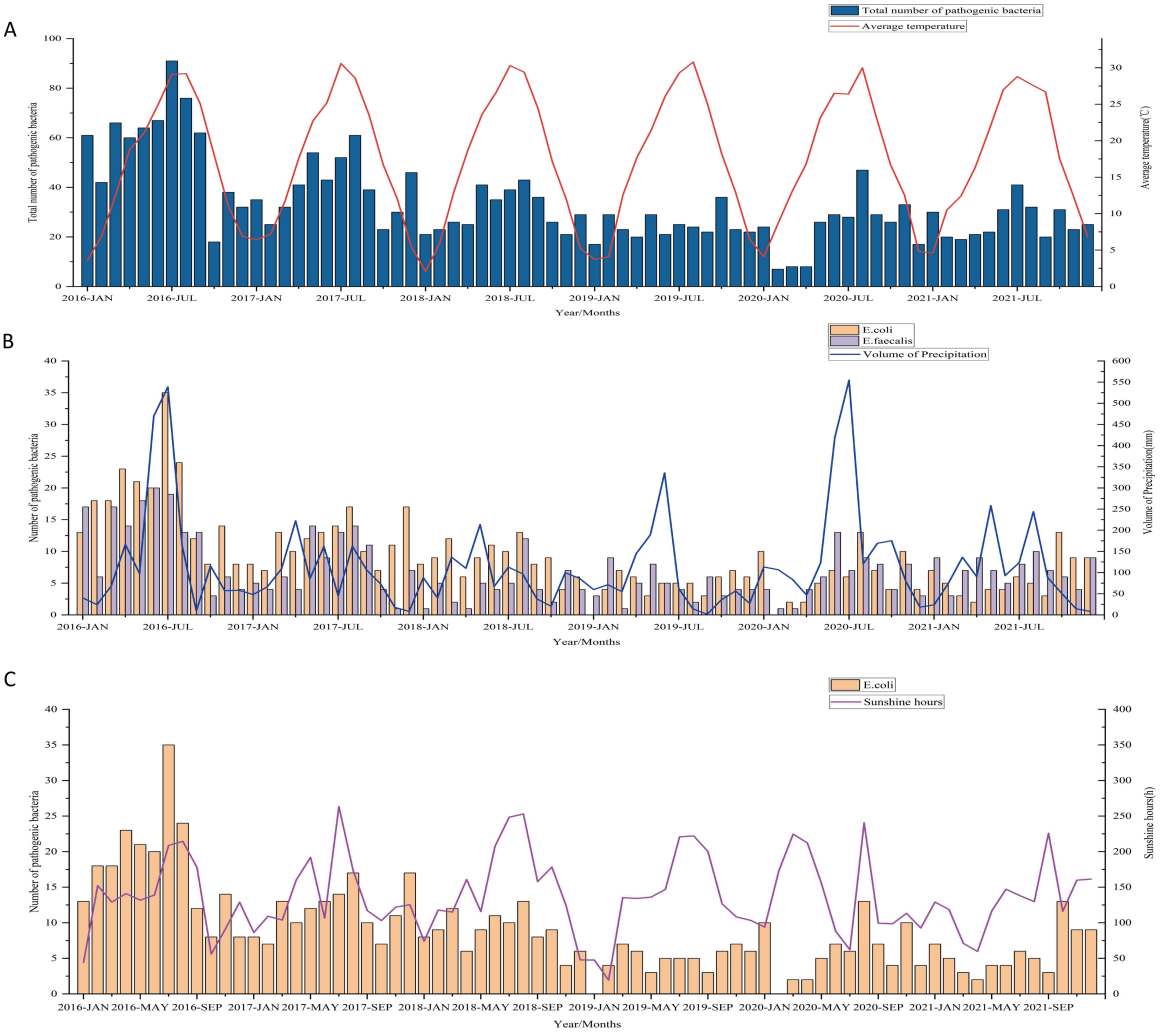
**(A)** Correlation between the total number of uropathogens detected and the monthly average temperature from 2016 to 2021. **(B)** Association between the number of *E. coli* and *E. faecalis* isolates and precipitation volume. **(C)** Relationship between the number of *E. coli* isolates and monthly sunshine hours.

Considering the correlation between various meteorological factors (co-linearity), multiple linear stepwise regression analysis were conducted with the total number of pathogenic bacteria detected per month and five main pathogens (*E. coli, E. faecium, E. faecalis, K. pneumoniae, and P. aeruginosa*) as dependent variables and monthly sunshine hours, average temperature, relative humidity, and precipitation volume as independent variables. Furthermore, considering the potential impact of seasons on the results, we simultaneously included the seasonal factor as an independent variable in the regression analysis. The total number of pathogenic bacteria was found to be significantly correlated with the monthly average temperature (*p* < 0.05, adjusted *R*^2^ = 0.114) (Table 2, Figure 4A). Interestingly, the number of pathogenic bacterial isolates was notably higher in most months of 2016 compared to subsequent years. From 2016 to 2021, we observed a consistent pattern where the monthly number of pathogenic bacteria increased with rising temperatures and decreased with falling temperatures, with cases predominantly concentrated during high-temperature seasons each year. In particular, the detection of *E. coli* showed significant correlations with both precipitation volume and sunshine hours. Higher precipitation was associated with increased detection of *E. coli* (standardized coefficient = 0.281, *p* = 0.015), with the monthly number of *E. coli* isolates rising as precipitation increased and decreasing with lower precipitation levels. Similarly, detection of *E. faecalis* also exhibited a significant correlation with the volume of precipitation (standardized coefficient = 0.387, *p* = 0.001) (Table 2, Figure 4B). This suggests a potential link between rainfall and bacterial contamination. Additionally, more sunshine hours were positively correlated with *E. coli* detection (standardized coefficient = 0.24, *p* = 0.037) (Table 2, Figure 4C).

**Table 2:** Multiple linear stepwise regression analysis model for correlation between monthly detections of major pathogenic bacteria and monthly mean values of meteorological factors.

Pathogen	Model summary		Correlation coefficients		
	Model significance (ANOVA)	Adjusted R2	Meteorological factor	Standard coefficient	*p* value
Total number of pathogenic bacteria	*p* < 0.05	0.114	Sunshine hours (h)	0.016	0.911
			Average temperature (°C)	0.356	0.002
			Relative humidity (%)	0.037	0.744
			Volume of Precipitation (mm)	0.169	0.171
			season	0.030	0.831
*E. coli*	*p* < 0.05	0.102	Sunshine hours (h)	0.24	0.037
			Average temperature (°C)	0.101	0.430
			Relative humidity (%)	−0.104	0.428
			Volume of Precipitation (mm)	0.281	0.015
			Season	−0.036	0.772
*E. faecium*	*p* = 0.766	/	/	/	/
*E. faecalis*	*p* < 0.05	0.138	Sunshine hours (h)	0.132	0.143
			Average temperature (°C)	0.210	0.084
			Relative humidity (%)	−0.077	0.538
			Volume of Precipitation (mm)	0.387	0.001
			Season	−0.090	0.449
*K. pneumoniae*	*p* = 0.469	/	/	/	/
*P. aeruginosa*	*p* = 0.713	/	/	/	/

### 3.5 Resistance of the main uropathogens to common antibiotics

**Figure 5:**
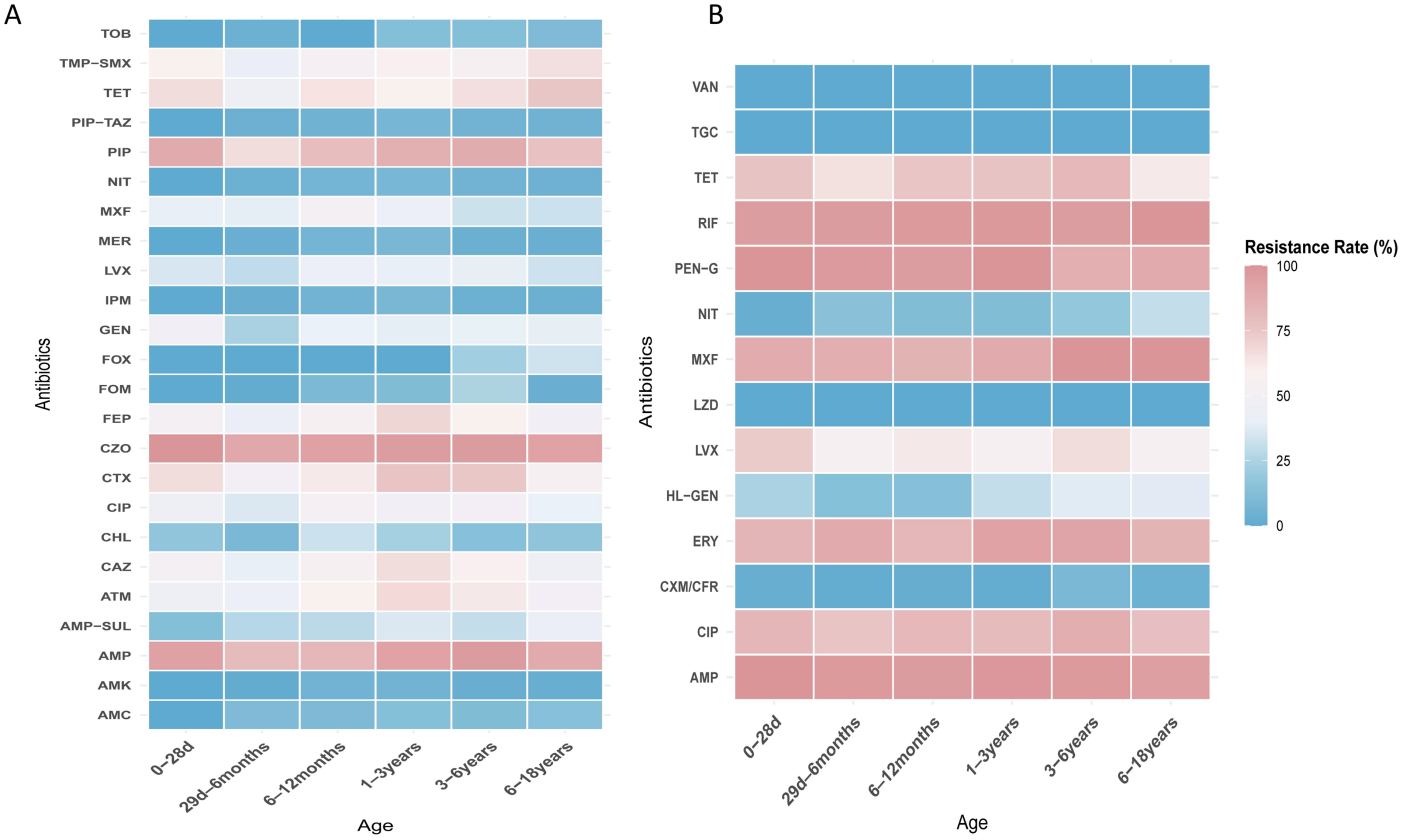
**(A)** Heat map of antibacterial resistance rates of *E. coli* across different age groups. **(B)** Heat map of antibacterial resistance rates of *E. faecium* across different age groups.

**Figure 6:**
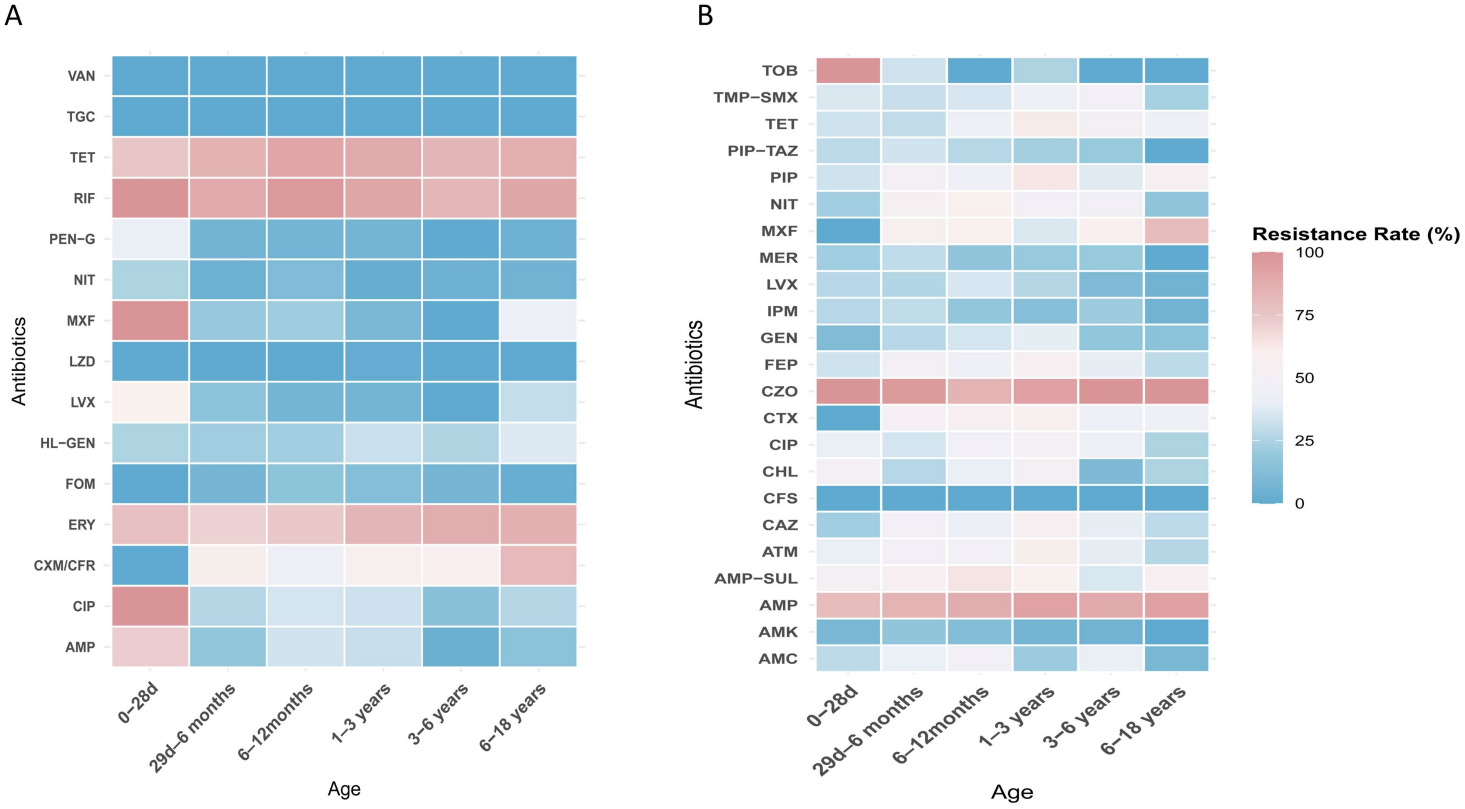
**(A)** Heat map of antibacterial resistance rates of *E. faecalis* across different age groups. **(B)** Heat map of antibacterial resistance rates of *K. pneumoniae* across different age groups.

*E. coli*, *E. faecium*, *E. faecalis*, and *K. pneumoniae* were the top four species detected in all age categories. Their antibiotic resistance rates based on different age categories are shown in Figures 5, 6. The resistance rates of *E. coli* to amoxicillin–clavulanic acid, piperacillin–tazobactam, cefoperazone-sulbactam, amikacin, fosfomycin and nitrofurantoin were low in all age categories, while that to ampicillin–sulbactam was lower in the ≤28-day age group than that in other age groups. Specifically, the resistance rate to fosfomycin in 3–6 years group was higher than other groups. Similarly, *E. coli* exhibited lower resistance rates to chloramphenicol and gentamicin in the 29-day–6-month age group. In contrast, the 1–3-year age group showed elevated resistance rates for cefotaxime (76.2%), ceftazidime (66.7%), cefepime (69.2%), and aztreonam (67.8%). In general, the resistance rates of *E. coli* against second-or third-generation cephalosporins were consistently high (surpassing 40%) across all age categories (Figure 5A). *E. faecium* exhibited no resistance to linezolid, vancomycin, and tigecycline across all age groups. Among the tested antibiotics, *E. faecium* demonstrated relatively lower resistance rates to high-level gentamicin in the 29-d–6-month and 6–12-month age groups, with 13.6 and 13.4% rates, respectively, while a higher resistance to nitrofurantoin was observed in the 6–18-year age group. Notably, *E. faecium* displayed comparatively higher resistance rates to tetracycline, fluoroquinolones, erythromycin, ampicillin, and penicillin (Figure 5B). *E. faecalis* did not demonstrate any resistance to linezolid, vancomycin, and tigecycline across all age categories. Relatively low resistance levels to fosfomycin, penicillin G, and nitrofurantoin were observed in most age groups, except for the ≤28-day group. Notably, high resistance rates against fluoroquinolones were observed in both the ≤28-day and 6–18-year age groups (Figure 6A). High resistance rates to ampicillin, ampicillin–sulbactam, and cefazolin against *K. pneumoniae*, ranging from 35.7 to 100%, were observed in all age groups. *K. pneumoniae* exhibited lower resistance rates to the aminoglycoside antibiotics, including amikacin, and gentamicin, in the 3–6-year and 6–18-year age groups. Additionally, *K. pneumoniae* displayed complete sensitivity to cephalosporin–sulbactam across all age groups. Conversely, higher resistance rates against third-generation cephalosporins, such as cefotaxime and ceftazidime, were observed in several age groups, including 49.5 and 51.6% in the 29-d–6-month age group, 55.6 and 56.2% in the 1–3-year age group, and 38.9 and 45.5% in the 3–6-year age group (Figure 6B).

## 4 Discussion

UTIs are the most common bacterial infections in children. However, their clinical presentation can vary significantly (22). The use of urine culture is still considered the gold standard for the diagnosis of UTIs (23). This research is the first large epidemiological study to systematically investigate the association between meteorological factors and the pathogens causing UTIs in a 6-years retrospective cohort of 2,441 pediatric patients with UTIs. Our findings reveal interesting results through multiple linear stepwise regression models, indicating positive correlations between the total number of pathogenic bacteria (especially, *E. coli*, and *E. faecalis*) causing UTIs and the average monthly temperature, precipitation, and hours of sunshine.

Our study reveals that temperature affects the occurrence of UTIs in children. The significant correlation between the total number of pathogenic bacteria and the monthly average temperature suggests that warmer weather may contribute to the proliferation and survival of these bacteria. Dehydration due to warmer weather leading to lower urine output has been proposed as a possible reason for the seasonality of UTIs (24–26). Furthermore, warmer weather may have other effects that increase the risk of UTIs. For instance, bacterial burden near the urethral opening tends to increase during warmer weather (15), as does bacterial skin colonization (27). The positive correlation between the UTIs caused by *E. coli* and *E. faecalis* with precipitation can be attributed to several factors. As reported in the literature (28), *E. faecalis* exhibits stronger environmental adaptability compared to other intestinal bacteria, such as *E. faecium.* It can survive for extended periods in aquatic or moist environments and is particularly resilient to various environmental stressors, including drought, high temperatures, and low oxygen conditions. This enhanced survival ability makes *E. faecalis* more likely to persist in the environment during heavy rainfall, increasing its potential for transmission. Increased precipitation can lead to higher concentrations of *E. coli* in urban streams and may wash fecal matter into storm drains and environmental water (29–31), thereby increasing children’s exposure and risk of developing UTIs.

The positive correlation between the *E. coli* detection count in the urinary tract and sunshine hours may be attributed to the extended outdoor activity time for children during longer daylight periods. Additionally, in China, the common practice of using diapers for children could potentially contribute to this correlation. Increased sunshine hours may result in more frequent and prolonged diaper usage, creating favorable conditions for the proliferation of *E. coli* and potentially increasing the risk of UTIs. Moreover, extended outdoor playtime in sunny weather may lead to increased sweating, and children may not adequately rehydrate. This can result in more concentrated urine, which allows microbes to remain in the urinary tract for longer periods, further increasing the risk of UTIs. Additionally, factors such as reduced hygiene practices during outdoor activities may contribute to the increased risk of UTIs. However, further research is needed to validate these hypotheses and establish a direct causal relationship. The climate in Wuhan is known for its scorching heat, earning it the nickname “furnace.” This is especially true during the months of May to July when the city experiences intense summer heatwaves. Consequently, there is a likelihood of increased usage of recreational water facilities such as swimming pools and water sprays. Children have a preference for water play, which undoubtedly raises the risk of UTIs.

In our study, the overall positive rate of pathogen detection from midstream urine samples was 7.9%, with a gradual decline over the years, from 677 positive samples in 2016 to 315 in 2021. Several factors may contribute to this trend. First, the decline in birth rates in China, from 18.83 million in 2016 to 9.02 million in 2023 (32), likely led to fewer pediatric patients seeking medical care, reducing the overall number of urine samples collected. Second, improvements in hygiene practices and increased parental awareness regarding children’s health, particularly in preventing urinary tract infections, may have contributed to a reduction in infection rates. These factors likely combined to produce the observed decrease in pathogen detection rates, though further studies would be needed to explore the underlying causes in more detail. The 29-day to 6-month age group exhibited the highest positive detection rate for UTIs. Several factors likely contribute to this trend. In Chinese tradition, infants under 1 month receive special care with heightened hygiene, which may reduce UTI risk. However, after 1 month, increased diaper use becomes more common, creating a warm, moist environment that may promote bacterial growth, raising the risk of UTIs (33). Additionally, the renal system of infants in this age group is still developing, making them more susceptible to infections. After 6 months of age, many parents begin using techniques such as creating water flow sounds or whistling to encourage independent urination, which leads to a reduction in diaper use and may help decrease the risk of UTIs (34). These physiological and cultural factors likely explain the higher detection rate observed in this age group.

Our study demonstrated *E. coli* as the main causative agent of UTIs among the children of Wuhan (27.1%), followed by *E. faecalis* (24.6%) and *E. faecalis* (20.7%). Other studies have indicated *E. coli*, *K. pneumoniae*, and *P. aeruginosa* to be major pathogens (35–37). Notably, the detection rate of *E. coli* as the principal UTI causative agent varies significantly across regions. A study from Nanjing, China, reported an occurrence rate of 22.32% for *E. coli* (38), whereas research by Demir et al. found a considerably higher detection rate of 58.9% (39). Similarly, Alsubaie et al. documented an even higher prevalence of 72.6% (40). These discrepancies in study findings may be attributed to regional variations, differences in environmental factors, variations in population demographics, as well as socioeconomic disparities and differences in healthcare access. Regions with better healthcare infrastructure and higher socioeconomic status may have more effective prevention and treatment measures, leading to lower UTI prevalence, while areas with limited access to healthcare and poorer sanitation may face higher infection rates due to delayed treatment, inadequate hygiene, and limited health resources.

The observed increase in the detection rates of *E. faecium* may be due to its ability to persist in harsh environments, as it can survive for months in aquatic settings and years on dry surfaces, making it difficult to eradicate in hospital environments. Additionally, *E. faecium* can adapt rapidly to new antibiotics, leading to increasing resistance over time. These factors likely contribute to its rising prevalence in clinical isolates, posing challenges for infection control and treatment (41). The detection rate of *E. coli* in UTIs gradually decreased from 31.6% in 2016 to 21.9% in 2021. This decline may be attributed to several factors, including the increased use of non-antibiotic therapies, such as probiotics and cranberry supplements, which help reduce the need for antibiotics and lower recurrence rates, thus further contributing to the decline in *E. coli* infections (42). Additionally, our study observed a rise in the prevalence of other pathogens, including Gram-positive bacteria and fungi (as indicated by the higher detection rates of *Enterococcus*). This suggests a shift in the microbial landscape of UTIs, which could explain the relative decline of *E. coli* in these infections. These factors, together, have likely reshaped the epidemiology of UTIs, leading to the observed decrease in *E. coli* detection rates.

Our findings also revealed interesting patterns regarding the predominance of specific pathogens in different age groups and gender differences in pathogen distribution. Children aged 0–1 year were the major demographics at risk of infection by *E. faecium*, while those aged 3–18 years were the major demographics at risk of infection by *E. coli*. This observation aligns with prior studies. Shaki et al. characterized the etiological profile of UTIs in children under 2 years of age and demonstrated that the proportion of *E. coli* increased with age, while the proportion of *Enterococcus* spp. decreased (43). Similarly, Lei Huang et al. reported that *E. faecium* predominated in younger age groups, such as newborns, whereas *E. coli* was more commonly isolated in older children (44). The predominance of *E. faecium* in this group may be attributed to several factors, including immune system immaturity, the pathogen’ s ability to persist in harsh environments, and its rapid adaptation to new antibiotics, all of which contribute to its higher detection rates in younger children.

Significant gender variations were also noted. *E. faecium* exhibited a higher prevalence in girls than it did in boys, while *E. faecalis* showed a higher prevalence in boys. Similar gender disparities were observed for *K. aerogenes*, *P. mirabilis*, and *M. morganii*. These findings suggest that the distribution of certain pathogens may be influenced by gender-specific factors (45). Research indicates that anatomical differences between males and females may contribute to variations in pathogen distribution. As mentioned in the background section, girls have a shorter urethra and a smaller distance between the urethral and anal opening, which increases the risk of fecal bacteria, such as *E. faecium*, migrating to the urinary tract. Additionally, the biological differences in immune response between males and females could influence UTI outcomes (46). These factors likely make females more susceptible to certain pathogens, such as *E. faecium*. In contrast, studies have shown that *E. faecalis* is more prevalent in males than in females, which may be related to physiological differences (47, 48). Furthermore, the etiological profile of *E. coli* demonstrated an increasing trend in girls with advancing age, while it remained stable in boys. Additionally, both male and female newborns were predominantly affected by *E. faecium*, indicating its significance as a common pathogen in the early stages of life (12). These findings shed light on the intricate interplay between biological, anatomical and behavioral factors in UTI prevalence. In addition, our study demonstrated that resistance rates to ampicillin, sulfamethoxazole–trimethoprim, penicillin, tetracycline, fluoroquinolones, and cephalosporins were higher in all age groups. These findings suggest that the inappropriate use of these drugs, particularly in community settings, may contribute to the observed elevated resistance rates. It is important to consider the role of community-based antibiotic stewardship programs (ASPs) in mitigating these trends. Studies have shown that ASPs implemented in healthcare settings can lead to a reduction in inappropriate antibiotic use, decreased resistance rates, and fewer cases of multidrug-resistant organisms (49, 50). However, challenges such as the rise of online medical consultations and the simplified access to antibiotics through community pharmacies, which often lack effective prescription monitoring, continue to contribute to the overuse and misuse of antibiotics in the community. Therefore, continuous and expanded efforts are required at both clinical and community levels to improve antibiotic stewardship and combat the growing threat of antibiotic resistance. However, it is noteworthy that *E. coli* demonstrated resistance rates of <10% against carbapenems, piperacillin–tazobactam, and nitrofurantoin. Therefore, these antibiotics could be considered as suitable empirical treatment options for UTIs caused by *E. coli*. Additionally, *E. faecium* and *E. faecalis* showed no resistance to linezolid, vancomycin, tigecycline, and furazolidone across all age groups. Hence, these antibiotics are effective treatment options. Given the absence of resistance, clinicians can consider the selective use of these antibiotics for empirical therapy in cases where *E. faecium* and *E. faecalis* are identified as the causative pathogens of UTIs, particularly before the results of antibiotic susceptibility testing are available. Incorporating these antibiotics into treatment guidelines for empirical therapy could provide clinicians with a reliable and effective therapeutic strategy in managing these infections.

Resistance patterns may vary across different age groups, for example, *E. faecium* showed relatively lower resistance to high-level gentamicin in the 29-d–12-month age groups. *E. faecalis* exhibited high resistance rates to fluoroquinolones in both the ≤28-day and 6–18-year age groups. Therefore, it is important to consider the age group when selecting appropriate treatment options for enterococcal infections.

The novelty of this study lies in integrating the distribution of pathogens, age-related characteristics, antibiotic resistance, and the correlation with meteorological factors, providing a comprehensive understanding of uropathogenic infections in pediatric UTIs and their association with meteorological factors. This will contribute to a better understanding of the pathogenesis of pediatric UTIs and provide a scientific basis for developing more effective prevention and treatment strategies. Moreover, this study will provide valuable clinical guidance to pediatricians. By understanding the distribution of pathogens and antibiotic resistance in different age groups of children with UTIs, healthcare professionals can make better-informed decisions regarding appropriate antibiotic treatment and avoid the issues of antibiotic resistance resulting from overuse. Furthermore, investigating the association between meteorological factors and pediatric UTIs can help predict and address seasonal peaks and develop relevant preventive measures.

Our study has several limitations. First, the study was conducted in a single healthcare facility with pediatric patients mainly from Wuhan. Hence, we cannot generalize our findings to other populations or districts. Hence, future studies should consider multicenter and/or multipopulation-based approaches. Second, the study primarily focused on the association between meteorological factors and the prevalence of pathogenic bacteria. However, correlations with additional meteorological variables (e.g., wind speed, atmospheric pressure, and temperature variation) and their impact on major pathogens, as well as less prevalent pathogens, were not explored, which could provide a more comprehensive understanding of the findings. Future multicenter studies incorporating broader environmental and microbial datasets are warranted to enrich the study’s scope and conclusions. However, it did not explore the underlying mechanisms or causality. Therefore, further research is needed to elucidate the specific pathways through which weather conditions may influence the occurrence and spread of pediatric UTIs. Additionally, changes in pathogen distribution and antibiotic resistance patterns over time were not extensively explored. Longitudinal studies with extended follow-up periods would be valuable in monitoring temporal trends and assessing the effectiveness of interventions aimed at mitigating antibiotic resistance in pediatric UTIs. Also, a minority of patients originated from areas surrounding Wuhan, but the environmental data collected did not comprehensively cover these regions. Therefore, the associations identified between pathogen prevalence and environmental factors might contain certain inaccuracies, not fully reflecting the actual conditions experienced by these specific patients. We are planning to collaborate with other centers to conduct such studies, which will provide more robust evidence for developing targeted interventions.

Despite these limitations, our results address an important gap by examining the relationship between meteorological factors and pathogenic bacterial infections in pediatric UTIs. We provide valuable insights into the influence of environmental parameters on common pathogens such as *E. coli* and *E. faecalis*, which are leading causes of UTIs in children. Detailed justifications were provided for the observed correlations between temperature, sunshine hours, and precipitation volumes with specific bacterial populations. These parameters significantly affect bacterial survival, proliferation, and transmission through mechanisms such as temperature-mediated metabolic activity, precipitation-induced water source contamination, and prolonged outdoor exposure during sunny weather. This comprehensive analysis enhances the understanding of how meteorological factors interact with pediatric UTI pathogenesis and provides a scientific basis for future preventive and therapeutic strategies.

## Data Availability

The original contributions presented in the study are included in the article/Supplementary material, further inquiries can be directed to the corresponding authors.

## Glossary

*E. coli*: *Escherichia coli*
*E. faecalis*: *Enterococcus faecalis*
*E. faecium*: *Enterococcus faecium*
*K. pneumoniae*: *Klebsiella pneumoniae*
*S. aureus*: *Staphylococcus aureus*
*P. aeruginosa*: *Pseudomonas aeruginosa*
*E. cloacae*: *Enterobacter cloacae*
*K. oxytoca*: *Klebsiella oxytoca*
*K. aerogenes*: *Klebsiella aerogenes*
*P. mirabilis*: *Proteus mirabilis*
*M. morganii*: *Morganella morganii*
IPM: Imipenem
MER: Meropenem
AMK: Amikacin
FOM: Fosfomycin
NIT: Nitrofurantoin
TOB: Tobramycin
FOX: Cefoxitin
CHL: Chloramphenicol
CIP: Ciprofloxacin
LVX: Levofloxacin
MXF: Moxifloxacin
ATM: Aztreonam
GEN: Gentamicin
FEP: Cefepime
CAZ: Ceftazidime
CTX: Cefotaxime
TET: Tetracycline
AMP: Ampicillin
PIP: Piperacillin
CZO: Cefazolin
LZD: Linezolid
VAN: Vancomycin
TGC: Tigecycline
ERY: Erythromycin
RIF: Rifampin
PEN-G: Penicillin G
AMP: Ampicillin
CFS: Cefoperazone-sulbactam
PIP-TAZ: Piperacillin-tazobactam
AMC: Amoxicillin-clavulanic acid
AMP-SUL: Ampicillin-sulbactam
TMP-SMX: Trimethoprim-sulfamethoxazole
CXM/CFR: Cefuroxime/Cefadroxil
HL-GEN: High-level Gentamicin
