# Supplementary material for: Influence of meteorological and environmental factors on pediatric urinary tract infections: insights from a 6-year retrospective study in Central China
Source: Front Public Health. 2025 Feb 13;13:1512403. doi: 10.3389/fpubh.2025.1512403 (PMC11864906; doi:10.3389/fpubh.2025.1512403)
Supplement: Supplementary file 1 [file Table_1.docx]

Supplementary Table 1. Distribution of main pathogens based on the gender.

| **Pathogenic bacteria** | **Male** | | **Female** | | ***p*-value** |
| --- | --- | --- | --- | --- | --- |
|  | ***n*** | **Percentage (%)** | ***n*** | **Percentage (%)** |  |
| *E. coli* | 331 | 27.8 | 323 | 26.5 | 0.452 |
| *E. faecium* | 204 | 17.1 | 388 | 31.8 | <0.01 |
| *E. faecalis* | 275 | 23.1 | 225 | 18.4 | <0.05 |
| *K. pneumoniae* | 114 | 9.6 | 95 | 7.8 | 0.116 |
| *P. aeruginosa* | 37 | 3.1 | 30 | 2.5 | 0.33 |
| *E. cloacae* | 26 | 2.2 | 24 | 2 | 0.706 |
| *K. oxytoca* | 27 | 2.3 | 18 | 1.5 | 0.149 |
| *K. aerogenes* | 26 | 2.2 | 11 | 0.9 | <0.05 |
| *P. mirabilis* | 22 | 1.8 | 11 | 0.9 | <0.05 |
| *M. morganii* | 23 | 1.9 | 6 | 0.5 | <0.01 |

Supplementary Table 2. Comparison of the etiological profiles of boys and girls across different age categories.

|  | **≤28 d** | | **29 d–6 months** | | **6–12 months** | | **1–3 years** | | **3–6 years** | | **6–18 years** | |
| --- | --- | --- | --- | --- | --- | --- | --- | --- | --- | --- | --- | --- |
| **Pathogenic**  **bacteria** | **Males**  **(*n* = 54)** | **Females**  **(*n* = 60)** | **Males**  **(*n* = 413)** | **Females**  **(*n* = 386)** | **Males**  **(*n* = 200)** | **Females**  **(*n* = 208)** | **Males**  **(*n* = 204)** | **Females**  **(*n* = 224)** | **Males**  **(*n* = 163)** | **Females**  **(*n* = 165)** | **Males**  **(*n* = 158)** | **Females**  **(*n* = 176)** |
| *E. coli* | 16.7% | 13.3% | 33.4% | 14.5% | 23.0% | 20.7% | 28.9% | 27.7% | 24.5% | 42.4% | 24.1% | 48.3% |
| *E. faecium* | 46.3% | 55.0% | 16.0% | 36.8% | 28.0% | 39.4% | 14.7% | 35.3% | 9.2% | 15.8% | 9.5% | 13.1% |
| *E. faecalis* | 14.8% | 5.0% | 23.7% | 27.5% | 21.5% | 21.2% | 19.6% | 14.7% | 28.2% | 7.9% | 26.6% | 13.6% |
| *K. pneumoniae* | 7.4% | 13.3% | 11.6% | 12.2% | 13.0% | 4.3% | 7.8% | 6.3% | 5.5% | 6.1% | 7.0% | 4.0% |
| *P. aeruginosa* | 0.0% | 0.0% | 0.5% | 0.3% | 1.5% | 2.9% | 3.9% | 2.2% | 7.4% | 5.5% | 7.6% | 5.1% |
| *E. cloacae* | 5.6% | 3.3% | 2.2% | 1.8% | 2.0% | 1.0% | 0.5% | 2.7% | 2.5% | 2.4% | 3.2% | 1.7% |
| *K. oxytoca* | 3.7% | 1.7% | 3.9% | 1.6% | 1.0% | 2.4% | 1.0% | 1.3% | 3.1% | 1.2% | 0.6% | 0.0% |
| *K.aerogenes* | 0.0% | 1.7% | 2.9% | 0.8% | 1.0% | 0.5% | 2.5% | 0.9% | 1.2% | 1.8% | 2.5% | 1.1% |
| *P. mirabilis* | 0.0% | 0.0% | 0.2% | 0.0% | 1.5% | 1.4% | 2.9% | 0.9% | 3.7% | 2.4% | 3.8% | 1.1% |
| *M. morganii* | 0.0% | 0.0% | 0.0% | 0.0% | 1.5% | 0.0% | 4.9% | 0.4% | 3.7% | 1.8% | 2.5% | 1.1% |
| *C. freundii* | 1.9% | 0.0% | 2.2% | 0.5% | 1.0% | 0.5% | 0.5% | 0.9% | 1.2% | 0.0% | 0.0% | 0.6% |
| *C. albicans* | 1.9% | 0.0% | 0.5% | 0.8% | 0.5% | 1.4% | 0.5% | 0.0% | 1.2% | 1.2% | 1.3% | 2.3% |
| *E. gallinarum* | 0.0% | 1.7% | 0.2% | 0.5% | 0.0% | 1.0% | 1.0% | 1.3% | 0.0% | 0.0% | 0.0% | 0.6% |
| *P. vulgaris* | 0.0% | 0.0% | 0.0% | 0.0% | 0.5% | 0.0% | 3.4% | 0.0% | 1.8% | 0.0% | 0.6% | 0.0% |
| *E. raffinosus* | 0.0% | 0.0% | 0.0% | 0.5% | 0.0% | 0.5% | 0.0% | 0.9% | 0.0% | 1.2% | 1.3% | 0.0% |
| *A. baumannii* | 0.0% | 0.0% | 0.2% | 0.3% | 0.0% | 0.0% | 1.0% | 0.9% | 0.0% | 0.6% | 0.0% | 1.1% |
| *S. aureus* | 0.0% | 0.0% | 0.2% | 0.0% | 0.0% | 0.0% | 0.5% | 0.0% | 1.2% | 1.2% | 1.3% | 0.0% |
| *E. avium* | 0.0% | 0.0% | 0.0% | 0.3% | 0.5% | 0.0% | 0.5% | 0.9% | 0.0% | 0.6% | 0.0% | 0.0% |
| *C. tropicalis* | 0.0% | 0.0% | 0.0% | 0.0% | 0.0% | 0.0% | 0.0% | 0.4% | 0.0% | 0.6% | 0.0% | 1.7% |
| *C. parapsilosis* | 1.9% | 1.7% | 0.0% | 0.0% | 0.0% | 0.0% | 0.0% | 0.0% | 0.0% | 0.0% | 1.3% | 0.0% |
| Others | 0.0% | 3.3% | 2.2% | 1.8% | 3.5% | 2.9% | 5.9% | 2.2% | 5.5% | 7.3% | 7.0% | 4.5% |
